# Inhibition of MUC1‐C regulates metabolism by AKT pathway in esophageal squamous cell carcinoma

**DOI:** 10.1002/jcp.27863

**Published:** 2018-12-06

**Authors:** Xin GongSun, YongQiang Zhao, Bin Jiang, ZhongWei Xin, Mo Shi, Liang Song, QiMing Qin, Qiang Wang, XiangYan Liu

**Affiliations:** ^1^ Department of Thoracic Surgery Shandong Provincial Hospital Affiliated to Shandong University Jinan, Shandong China; ^2^ Department of Thoracic Surgery Laiwu City People’s Hospital Laiwu, Shandong China

**Keywords:** AKT, ESCC, GO‐203, metabolism, MUC1‐C, TIGAR

## Abstract

Esophageal squamous cell carcinoma (ESCC) is one of the most common digestive tumors worldwide. The Mucin 1 (MUC1) heterodimeric protein has been confirmed that is overexpressed in ESCC and induced adverse outcomes. However, the detailed mechanism(s) remained challenging. So, we investigated the relationship between MUC1‐C and metabolism in ESCC cells. In the results, TP53‐induced glycolysis and apoptosis regulator (TIGAR) was overexpressed and correlative with MUC1‐C positively in ESCC tissue. Targeting MUC1‐C inhibits AKT–mTORC–S6K1 signaling and blocks TIGAR translation. We found that the inhibitory effect of GO‐203 on TIGAR was mediated by inhibition of AKT–mTOR–S6K1 pathway. The findings also demonstrated that the suppressive effect of GO‐203 on TIGAR is related to the decrease of glutathione level, the increase of reactive oxygen species and the loss of mitochondrial transmembrane membrane potential. In xenograft tissues, GO‐203 inhibited the growth of ESCC cells and lead to the low expression of transmembrane C‐terminal subunit (MUC1‐C) and TIGAR. This evidence supports the contention that MUC1‐C is significant for metabolism in ESCC and indicated that MUC1‐C is a potential target for the treatment of ESCC.

## 1 INTRODUCTION

Esophageal squamous cell carcinoma (ESCC) is one of the most common digestive tumors in the world, especially in developing countries (Torre et al., 2015). Surgical resection is the standard treatment for ESCC, but, it is not that effective for patients who want to survive for a long time (Mariette, Piessen, & Triboulet, 2007; Mizoguchi et al., 2014). The biological characteristics of ESCC are growing malignantly and metastasizing early, which lead to poor prognosis. Therefore, to explore the molecular pathological mechanism of ESCC is of great significance in diagnosis and treatment.

Mucin 1 (MUC1) is a transmembrane heterodimer glycoprotein which is aberrantly overexpressed in ESCC (Ye et al., 2011). And it had been reported that the overexpression is related to the characteristics of a tumor (Nath & Mukherjee, 2014). MUC1 is processed by autocleavage into two subunits, the extracellular N‐terminal subunit and the transmembrane C‐terminal subunit (MUC1‐C). These two subunits form a stable heterodimeric complex at the cell membrane (Kufe, 2009). MUC1‐C consists of an intrinsically disordered 72 amino acid cytoplasmic domain (MUC1‐CD), which is phosphorylated by different kinases and interacts with various effectors associated with transformation (D.W. Kufe, 2013; Huang et al., 2005). Importantly, MUC1‐C interacts with diverse effectors such as β‐catenin (Yamamoto, Bharti, Li, & Kufe, 1997), IKK β (Ahmad et al., 2007), and NFkB p65 (Ahmad et al., 2009), which have been connected to transcription. MUC1‐C inhibits mitochondrial transmembrane potential (MMP) loss induced by stress through the incorporation of the mitochondrial outer membrane (Ren et al., 2006). Overexpression of MUC1 in human cancers blocks the effects of DNA damage agent (Ren et al., 2004), reactive oxygen species (ROS; Yin, Huang, & Kufe, 2004; Yin, Li, Ren, Kuwahara, & Kufe, 2003) and hypoxia (Yin, Kharbanda, & Kufe, 2007) on cell apoptosis and necrosis. The MUC1‐C cytoplasmic domain contains a CQC motif that is necessary for MUC1‐C homodimerization and function (Leng et al., 2007; Raina et al., 2012). Therefore, GO‐203, cellular penetrating peptides, have been developed to target the MUC1‐C CQC motif, blocking the activation of various MUC1‐C‐mediated pathways (Raina et al., 2011).

Proliferation and metastasis of the tumor are accompanied by changes in metabolism and secondary microenvironment. TP53‐induced glycolysis and apoptosis regulator (TIGAR) is considered as a metabolic index. Previous studies have shown that high levels of TIGAR expression are closely associated with poor clinical outcomes in patients with multiple types of cancer including chronic lymphocytic leukemia (Hong et al., 2016), invasive breast cancer (Won et al., 2012), Stages II and III colorectal cancer (Alkhayal et al., 2016), nasopharyngeal carcinoma (Wong et al., 2015; Zhao et al., 2016), and non‐small‐cell lung cancer (Shen et al., 2018).

The phosphoinositide 3‐kinase (PI3K), AKT (also known as protein kinase B, PKB) and mammalian target of rapamycin (mTOR), dominate various indications of cancer including cell cycle, survival, metabolism, motility, and genomic instability. PI3K–AKT signaling pathway regulates the translation in the upstream of rapamycin complex 1 (mTORC1) target. mTORC1 energizes 40S ribosomal protein S6 kinases (S6Ks) that promote cap‐dependent translation and thus upgrade the eIF4A RNA helicase activity (Sonenberg & Hinnebusch, 2009). S6Ks lead to degradation of suppressor programmed cell death protein 4 (PDCD4), which is an eIF4A inhibitor in cancer cells (Dorrello et al., 2006). eIF4A sets up translation by unraveling highly organized 5′ untranslated regions in messenger RNA (mRNA), just like the encoding cyclin D1 and MYC (Rogers, Komar, & Merrick, 2002). Tumor cells can induce eIF4A RNA helicase to regulate intracellular transcriptional and translation through the AKT–mTORC pathway.

Our previous study has demonstrated that the MUCI‐C is overexpressed in ESCC tissue with an important function in hallmarks of ESCC such as proliferation, metastasis, and antiapoptosis (Xin et al., 2018). And another report has described that effect of GO‐203 is related to downregulation of the AKT–mTOR pathway and inhibition of cap‐dependent translation of TIGAR protein (Ahmad et al., 2017). However, the detailed mechanism of MUC1‐C in ESCC remains unclear. In the study, we had explored the effects of MUC1‐C in metabolism and the mechanism in human ESCC cells.

## 2 MATERIALS AND METHODS

### 2.1 Patient

Twenty pairs of ESCC tissues and paracancerous esophageal tissues (>5 cm from the margin of the tumor) were collected from the Department of Thoracic Surgery at Shandong Provincial Hospital Affiliated to Shandong University from October 2016 to January 2017. All patients met the following conditions: (a) pathological diagnosis of ESCC; (b) no preoperative chemotherapy or radiotherapy; (c) the incisal edge was proved to be as normal esophageal tissue by pathology. This study was approved by the Ethics Committee of Shandong Provincial Hospital Affiliated to Shandong University in accordance with the ethical guidelines of the Declaration of Helsinki. All participants had signed written informed consent.

### 2.2 Reagents and cell culture

Human ECA109 and KYSE150 ESCC cells were maintained in RPMI‐1640 medium supplemented with 10% fetal bovine serum (BI, Kibbutz Beit Haemek, Israel), 1% penicillin/streptomycin, and 1% l‐glutamine. The cells were treated with the MUC1‐C inhibitor peptide GO‐203 or control peptide CP‐2 (NGPeptide; China). Cells were treated with the PI3K inhibitor LY294002, AKT inhibitor GSK690693, and mTORC Inhibitor Rapamycin (Selleck Chemicals, Houston, TX).

### 2.3 Western blot analysis

All proteins were prepared as described (Xin et al., 2018) and analyzed by immunoblotting with anti‐p‐AKT, anti‐AKT and anti‐S6K (OmnimAbs, California), anti‐p‐S6K1, anti‐MUC1‐C, anti‐TIGAR, anti‐PDCD4 (Abcam, San Francisco, CA), and anti‐β‐actin (Boster, Wuhan, Hubei, China). Reactivity was quantified with horseradish peroxidase‐conjugated secondary antibodies and enhanced chemiluminescence detection system (Amersham Imager 600; General Electric, Fairfield, CT).

### 2.4 ROS measurement

The cells were made into suspensions and fully mixed with 10 μM DCFH‐DA (Beyotime, Shanghai, China), incubated at 37° for 25 min. Reactive oxygen was measured by flow cytometry.

### 2.5 Quantitative reverse‐transcriptase polymerase chain reaction

Whole‐cell RNA was abstracted using the RNAiso Plus (Takara Bio Inc., Kusatsu, Shiga, Japan) according to the manufacturer’s instructions. The complementary DNA samples were augmented by the SYBR Green quantitative PCR assay kit (Takara Bio Inc.) and the LightCycler480 PCR system(Applied Biosystems, Massachusetts). The primers were as follows: TIGAR, forward: 5′‐CTCCAGTGATCTCATGAG‐3′ and reverse 5′‐AGACACTGGCTGCTAATC‐3′; β‐actin, forward: 5′‐AGAGCCTCGCCTTTGCCGATCC‐3′ and reverse 5′‐ATACACCCGCTGCTCCGGGTC‐3′.

### 2.6 Determination of GSH levels

GSH can induce 5,5′dithiobis‐2‐nitrobenoic acid to transform into 2‐nitro‐5‐tiobenzoic acid, which absorbs maximum light at 412 nm. The specific experimental steps are operated according to the instructions by GSH content determination kit (Solarbio, Beijing, China). The absorbance was measured at 412 nm and calculation based on the standard curve of GSH.

### 2.7 MMP analysis

MMP was measured using the Mitochondrial Membrane Potential Assay Kit with JC‐1 (Beyotime) as described (Lu et al., 2015). The images were examined by confocal microscopy (Molecular Devices).

### 2.8 Immunohistochemistry

The expression of MUC1‐C and TIGAR was performed by the streptavidin‐peroxidase method. Patient tissues and xenograft tumor tissues were fixed with formalin and sectioned after paraffin embedded. Antigen was retrieved after dewaxing and hydration and then incubated with hydrogen peroxide. Tissue sections were incubated with rabbit anti‐MUC1‐C and anti‐TIGAR antibodies (1:100; Abcam) overnight at 4°C. Then marked these sections by a secondary biotinylated antibody (ZSGB Biotech, Beijing, China) and color development utilizes the diaminobenzidine (DAB) method. The images were examined using a fluorescence microscope (Olympus BX51; Olympus, Japan) and evaluated by two independent pathologists.

### 2.9 Apoptosis analysis

Cells were prepared according to the P‐phycoerythrin (PE) Annexin V apoptosis detection kit with 7‐amino‐actinomycin D (D7‐AAD; Becton Dickinson, New Jersey). After incubated using PE and 7‐AAD for 15 min, the cells were determined by ﬂow cytometry.

### 2.10 ESCC tumor xenograft in mice

Six‐to eight‐week‐old female BALB/c nu/nu mice were injected with 1 × 10^7^ ESCC ECA109 cells subcutaneously in the flank. When tumors reached ~150 mm^3^, the mice were pair matched into three groups of five mice each, and treated with (a) 15 mg/kg CP‐2 administered intraperitoneally daily, (b) 15 mg/kg GO‐203 administered intraperitoneally each day, (c) phosphate‐buffered saline (PBS; control vehicle). Recording tumors size every 4 days and weighed them after 20 days. Tumor volumes were calculated by using the formula *V* = *L* x *W* ^2^/2, where *L* and *W* are the largest and smallest diameters, respectively. The protocol was approved by the Animal Ethics Committee of Shandong Provincial Hospital Affiliated to Shandong University.

### 2.11 Statistical analysis

SPSS 20.0 (IBM, Armonk, NY) was used to create databases for data analysis. The quantitative data were expressed as a mean ± standard deviation. For two groups, two‐tailed Student’s *t* test was performed, and for three or more than three groups, one‐way analysis of variance was used. The relationship between MUC1‐C and TIGAR expression in patient tumors and xenograft tumor was calculated by correlation analysis. A value of *p* < 0.05 was selected before the experiments to indicate statistical significance.

## 3 RESULTS

### 3.1 TIGAR overexpresses in ESCC tissue and targeting MUC1‐C inhibits TIGAR translation in ESCC cells

**Figure 1:**
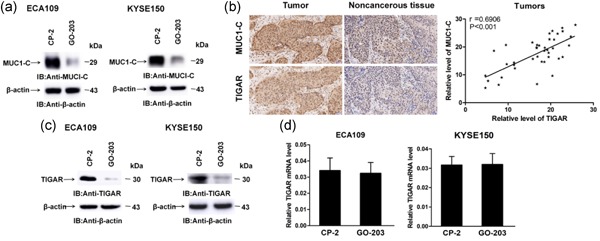
TIGAR overexpresses in ESCC tissue and targeting MUC1‐C inhibits TIGAR translation. (a) ECA109 and KYSE150 cells were treated each day with 5 μM GO‐203 or CP‐2 for 3 days, and then the expression of MUC1‐C was detected. (b) MUC1‐C and TIGAR expression in tumor tissues and noncancerous tissues from patients were shown by immunohistochemistry (×200; left). Correlation analysis of the relative level of MUC1‐C and TIGAR in tumors (right). (c) The expression of TIGAR in GO‐203 or CP‐2 treated ECA109 and KYSE150 cells. (d) TIGAR mRNA levels in ESCC cells were determined by qRT‐PCR. The relative TIGAR mRNA levels compared with that obtained for β‐actin as a control. ESCC: esophageal squamous cell carcinoma; mRNA: messenger RNA; MUC1‐C: transmembrane C‐terminal subunit; qRT‐PCR: quantitative reverse‐transcriptase PCR; TIGAR: TP53‐induced glycolysis and apoptosis regulator [Color figure can be viewed at wileyonlinelibrary.com]

The MUC1‐C expression was obviously inhibited by the treatment of ECA109 and KYSE150 cells with GO‐203, but inconspicuous in the control group (Figure 1a). The expression of MUC1‐C and TIGAR in tumor tissue and normal tissues were investigated using immunohistochemistry. Obvious staining was shown in the cytoplasm of tumor tissue but weak in normal esophageal tissue (Figure 1b; left), and the correlation of MUC1‐C and TIGAR was positive (Figure 1b; right). Targeting MUC1‐C has been reported to inhibit TIGAR at the protein level in hematologic malignancies (Yin, Kosugi, & Kufe, 2012; Yin, Kufe, Avigan, & Kufe, 2014) and colon cancer (Ahmad et al., 2017). In addition, the treatment of ECA109 and KYSE150 cells with GO‐203 lead to a low expression of TIGAR levels (Figure 1c). As mentioned, there was no significant change in TIGAR mRNA levels between the two treatment cells (Figure 1d). These results suggest that TIGAR is closely related to the ESCC and it is regulated effectively by MUC1‐C.

### 3.2 Targeting MUC1‐C inhibits AKT–mTORC–S6K1 signaling in ESCC cells

**Figure 2:**
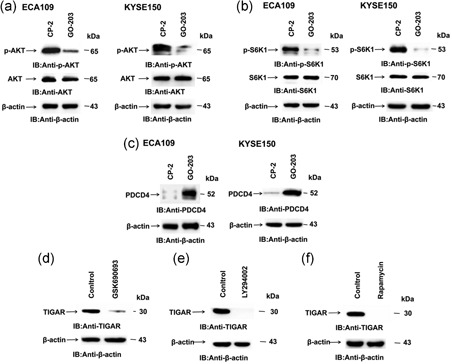
Targeting MUC1‐C downregulates AKT–mTOR–S6K1 translation pathway and the pathway is involved in TIGAR protein synthesis. (a–c) ECA109 and KYSE150 cells were treated each day with 5 μM GO‐203 or CP‐2 for 3 days, and GO‐203 inhibits the phosphorylation of AKT and S6K1 protein and then increases PDCD4 leave in ESCC cells. (d–f) TIGAR expression was detected in ECA109 cells which were treated with the PI3K inhibitor LY294002 (25 μM) and AKT inhibitor GSK690693 (10 μM) for 48 hr, mTORC inhibitor Rapamycin (100 nM) for 24 hr. ESCC: esophageal squamous cell carcinoma; MUC1‐C: transmembrane C‐terminal subunit; mTOR: rapamycin; PDCD4: programmed cell death protein 4; PI3K: phosphoinositide 3‐kinase; S6K1: ribosomal protein S6 kinases 1

The effect of GO‐203 on ECA109 and KYSE150 cells was related to the downregulation of p‐AKT (Figure 2a) and phosphorylation of S6k1 (Figure 2b). The activation of S6K1 leads to degradation of PDCD4, which increases the activity of elF4A. Activation of eIF4A RNA helicase participates in the initiation of translation. The GO‐203 treatment of ECA109 and KYSE150 cells promoted the expression of PDCD4 (Figure 2c), which is an inhibitor of eIF4A RNA helicase. These results proved that the MUC1‐C acts the function by regulating AKT–mTORC–S6K1 signaling in ECSS. To verify whether TIGAR translation could be affected by AKT–mTORC–S6K pathway, PI3K inhibitor LY294002, and AKT inhibitor GSK690693 were applied in ECA109 cells. Both inhibitors could significantly reduce TIGAR expression in ESCC cells (Figure 2d,e) which manifest TIGAR is the downstream target protein of the PI3K–AKT pathway. mTORC inhibitor rapamycin was used to detect TIGAR levels to confirm its potential role. Indeed, the treatment of ECA109 cells with rapamycin inhibited TIGAR levels (Figure 2f). These findings demonstrated that the MUC1‐C regulates TIGAR protein translation by the PI3K–AKT–S6K1 pathway.

### 3.3 Targeting MUC1‐C enhances ROS and inhibits GSH levels

**Figure 3:**
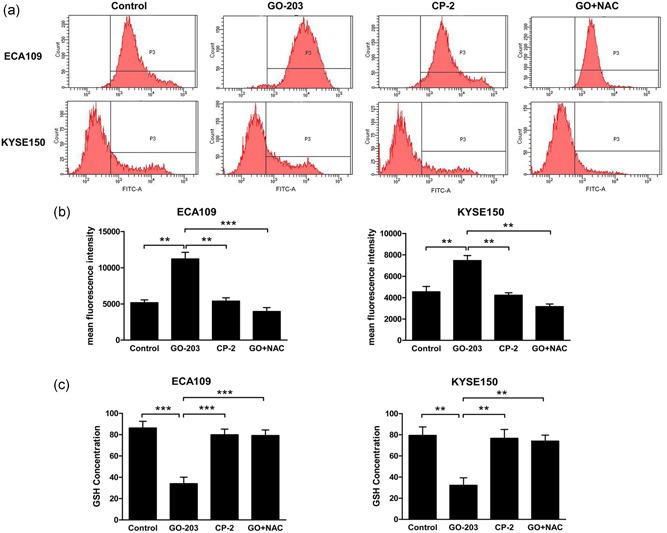
Inhibition of MUC1‐C disrupts the redox balance. (a–b), ECA109 and KYSE150 cells were treated each day with 5 μM GO‐203 or CP‐2 for 3 days, and 5 mM NAC was also added in GO‐203 treated cells. The ROS generation was measured using DCFH‐DA by flow cytometry, and mean fluorescence intensity was the symbol of ROS production. (c) Cells were prepared as previously mentioned in (a–b). The cells were analyzed for GSH levels. **p < 0.01; ***p < 0.001. MUC1‐C: transmembrane C‐terminal subunit; ROS: reactive oxygen species [Color figure can be viewed at wileyonlinelibrary.com]

As mentioned earlier, the disruption of redox equilibrium was blocked by MUC1‐C, and the silencing of MUC1‐C was related to the increase of ROS production (Yin et al., 2003). The GO‐203 treatment of ECA109 cells increases ROS production significantly compared with CP‐2 (Figure 3a,b). GSH can affect the metabolic process of cells by activating a variety of enzymes. GO‐203 shows a significant inhibitory effect on the GSH level of ECA109 and KYSE150 cells (Figure 3c). In addition, GO‐203 reduced GSH levels and induced ROS production return to the original level after the addition of N‐acetyl‐L‐cysteine (NAC). According to these results, inhibition of MUC1‐C leads to the production of ROS and consumption of GSH. In addition, both of these effects of GO‐203 were reversed by NAC.

### 3.4 Targeting MUC1‐C inhibits mitochondrial membrane potential and promotes apoptosis in ESCC cells

**Figure 4:**
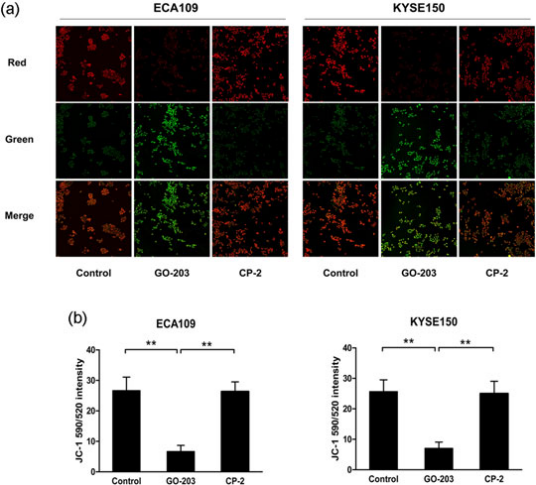
Targeting MUC1‐C decrease mitochondrial membrane potential. (a) ECA109 and KYSE150 cells were treated with GO‐203 and CP‐2 for 3 days then mitochondrial membrane potential (JC‐1) was measured by confocal microscopy (×100). Red fluorescence means normal cells. Green fluorescence cells indicate loss of mitochondrial membrane potential. (b) These results are expressed as JC‐1 590/520 intensity. **p < 0.01. MUC1‐C: transmembrane C‐terminal subunit [Color figure can be viewed at wileyonlinelibrary.com]

**Figure 5:**
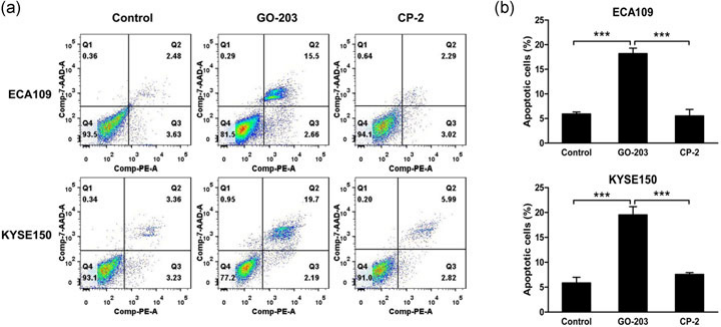
Inhibition of MUC1‐C promotes apoptosis. (a) The cells were treated with 5 μM GO‐203 or CP‐2 for 3 days and then been incubated with PE/7‐AAD and analyzed by flow cytometry. (b) The results are expressed as the percentage of apoptotic cells. ***p < 0.001. 7‐AAD: 7‐amino‐actinomycin D; MUC1‐C: transmembrane C‐terminal subunit; PE: P‐phycoerythrin [Color figure can be viewed at wileyonlinelibrary.com]

Redox balance is pivotal in metabolism, and its disruption can lead to loss of mitochondrial membrane potential loss and cell apoptosis (Yin et al., 2011). In the result, green fluorescence was increased in ECA109 and KYSE150 cells treated with GO‐203, which confirmed the reduction of mitochondrial membrane potential (Figure 4a,b). In the flow result, we find the apoptosis of ECA109 and KYSE150 cells that treated with GO‐203 increased signiﬁcantly, but not obvious in control CP‐2 groups (Figure 5a, B).

### 3.5 GO‐203 induces suppression of ESCC tumors in mice

**Figure 6:**
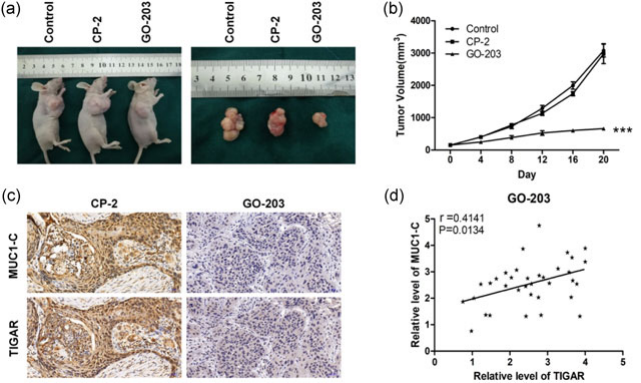
Targeting MUC1‐C regress tumor growth and TIGAR expression in vivo. (a) BALB/c nude mice were injected subcutaneously in the flank with 1 × 10^7^ ECA109 cells. The mice were pair matched when the tumors were ~150 mm^3^ in size. Treatment groups consisted of 5 mice injected intraperitoneally with PBS (vehicle control), 15 mg/kg GO‐203 or 15 mg/kg CP‐2 each day for 20 days. (b) Volume of tumors were performed as indicated every 4 days. (c) Xenograft tumor tissues were stained for MUC1‐C and TIGAR expression by immunohistochemistry (×200). (d) Relative levels of MUC1‐C and TIGAR with GO‐203 treatment were detected and analyzed by correlation analysis. **p < 0.01; ***p < 0.001. MUC1‐C: transmembrane C‐terminal subunit; PBS: phosphate‐buffered saline; TIGAR: TP53‐induced glycolysis and apoptosis regulator [Color figure can be viewed at wileyonlinelibrary.com]

Tumor xenografts of nude mice were established to study the GO‐203 function in vivo. The average volumes of tumors in the GO‐203‐treated mice were repressed obviously. Contrary to the antitumor activity of GO‐203, CP‐2 treatment has no effect on ECA109 tumor growth (Figure 6a,b). Tumor tissues of mice treated with GO‐203 and CP‐2 were stained for MUC1‐C and TIGAR. Consistent with previous results, both MUC1‐C and TIGAR levels in GO‐203‐treated mice were decreased substantially (Figure 6c), there was a positive correlation between MUC1‐C and TIGAR expression after treated with GO‐203 (Figure 6d). These results confirmed that targeting MUC1‐C inhibits ESCC tumor growth and TIGAR expression in vivo.

## 4 DISCUSSION

ESCC is one of the cancers with high incidence and high mortality. The main cause of poor prognosis is that ESCC metastasizes at the early stage (Martin, Herbert, & Hocevar, 2010). Gene therapy has become a new direction of cancer treatment. It is of great significance to search for the genes associated with ESCC and to clarify the mechanism of action in the treatment of ESCC.

MUC1 has been confirmed to be overexpressed in ESCC (Jing et al., 2015) and associated with its self‐renewal and tumorigenicity (Shi, Chen, Yang, & Liu, 2015). MUC1 mainly relies on the MUC1‐C subunit to function after translation. MUC1‐C interacts with multiple kinases and effectors owing to its special structure MUC1‐CD, and then participate in transformation (D.W. Kufe, 2013; Kufe, 2009). The MUC1‐C transforming function is dependent on homodimer formation, which is mediated by a CQC motif present in the cytoplasmic domain of MUC1‐C (Leng et al., 2007). MUC1‐C transmits signals as signal proteins in cells, but this function can be inhibited by GO‐203 (Raina et al., 2009). The inhibitor has been reported in inhibiting cell proliferation in vitro effectively of breast cancer (Raina et al., 2009), prostate cancer (Joshi et al., 2009), non‐small‐cell lung cancer (Bouillez et al., 2016), colorectal cancer cells (Takahashi et al., 2015), and certain hematologic malignancy (Yin et al., 2010). We had demonstrated that MUC1‐C is linked to the growth, metastasis, and other characteristics of ESCC cells (Xin et al., 2018). The specificity of GO‐203 on ESCC cells is notable, because of the binding to the CQC motif of MUC1‐C and inhibition of its function. In contrast, the CQC motif in CP‐2 has been changed to AQA motif, so CP‐2 does not inhibit MUC1‐C functioned in ESCC cells.

TIGAR was originally thought to be the transcriptional target of p53 (Bensaad et al., 2006). As a major tumor suppressor protein, the function of p53 is to inhibit the growth or survival of tumor cells by inducing apoptosis or senescence. What is more, growing studies have reported that p53 can also regulate metabolism (Vousden & Ryan, 2009), and TIGAR facilitates the activation of p53. The activity of TIGAR and other genes by p53 promotes an antioxidant response (Budanov, Sablina, Feinstein, Koonin, & Chumakov, 2004; Cosentino, Grieco & Costanzo, 2014), which helps cells to survive transient or low levels of stress, and has lately been attested to be significant in impeding malignant progression (Li et al., 2012). We testified that MUC1‐C and TIGAR are overexpressed in ESCC tissue and there is a positive correlation between them. The previous study had reported inhibition of MUC1‐C in hematological malignancies and colon cancer (Ahmad et al., 2017) downregulates TIGAR at the protein level (Yin et al., 2012, 2014). GO‐203 also reduced TIGAR protein expression in the treated ESCC cells, but there was no change in mRNA level. We verified that MUC1‐Cregulate TIGAR at the translation level but not at transcriptional level.

In recent years, research of PI3K–AKT–mTOR signaling pathway has got diverse achievements. This signaling pathway now is considered as a pivotal regulator in the activities of the cell cycle, cellular proliferation, growth, survival, protein synthesis, and glucose metabolism (Engelman, Luo, & Cantley, 2006; Vanhaesebroeck, Stephens, & Hawkins, 2012). AKT controls translation by activation of mTORC1 that results in the phosphorylation of p70S6 Kinase (Ma & Blenis, 2009). In turn, S6K phosphorylates and thereby induces the degradation of PDCD4, an inhibitor of eIF4A RNA helicase activity that regulates translation of proteins (Dorrello et al., 2006). This study confirms that the targeting MUC1‐C by GO‐203 inhibits phosphorylation of AKT and S6K in ESCC cells. PDCD4 is a downstream target of S6K1, which enhances protein synthesis to promote cell growth and survival. GO‐203 promotes PDCD4 expression, which is an inhibitor of elF4A helicase. According to these outcomes, GO‐203 regulates AKT–S6K1–elF4A signaling in ESCC cells. In our study, it shows that the treatment of ESCC cells with PI3K inhibitor LY294002 and AKT inhibitor GSK690693 both downregulated TIGAR proteins. Rapamycin, that is an inhibitor to mTORC1 also downregulated TIGAR protein level. These findings thus confirm that the MUC1‐C regulated TIGAR translation by AKT–S6K1–elF4A pathway.

TIGAR also reduces the content of ROS in cells by increasing the production of NADPH (Yin et al., 2012). NADPH can convert GSH oxide into reduced glutathione. With the GO‐203 treatment of ESCC cells, GSH decreased and ROS increased, which suggest that inhibition of MUC1‐C could deplete NADPH. Changes in redox equilibrium caused by GO‐203 significantly decreased the level of GSH. After the addition of NAC into GO‐203, it was reversed that GSH recovered and ROS decreased to the original level. The electron transport chain present in the mitochondrial inner membrane generates ROS. The previous research has confirmed that the MUC1‐C translocates to the mitochondrial outer membrane during the stress response, thus attenuating the reducing of mitochondrial membrane potential (Ren et al., 2004, 2006). Our findings have shown that inhibition of MUC1‐C leads to mitochondrial membrane potential loss. The inhibition of MUC1‐C induced disruption redox imbalance by GO‐203 was related to the loss of mitochondrial membrane potential. In cooperate with this reaction, GO‐203 induced apoptosis of ESCC cells. These results suggest that MUC1‐C plays an important role in the metabolism of ESCC cells.

Moreover, GO‐203‐treated ESCC tumors showed an evident slowing of growth in vivo. With a long time of the GO‐203 treatment, the volumes of xenograft tumor in nude mice were significantly reduced. In contrast, the control group, with CP‐2 or PBS, has no obvious inhibitory effects. The results suggest that GO‐203 is efficacious in inhibiting ESCC tumor proliferation in xenograft models. The effectiveness of GO‐203 in ESCC tumor xenografts can be related to the expression of MUC1‐C and TIGAR in human ESCC. In immunohistochemistry results, we also found that targeting MUC1‐C with GO‐203 downregulated TIGAR expression in xenograft tissues and which confirmed the correlation between MUC1‐C and TIGAR. Therefore, the effect of MUC1‐C on ESCC patients is associated with TIGAR.

This study supports that MUC1‐C is crucial in supporting the growth, survival, and metabolism of ESCC cells. Importantly, MUC1‐C is basically disordered and thereby has the capacity to serve as a substrate for multiple kinases and as a binding partner for diverse effectors of gene transcription. Such as, (a) the MUC1‐C cytoplasmic domain is phosphorylated by GSK3b and binds directly to β‐catenin, linking MUC1‐C to the WNT pathway (Yin et al., 2017); (b) MUC1‐C is also known to bind IKKβ (Ahmad et al., 2007) and Rel A p65 (Ahmad et al., 2009) and then participates constitutive NFκB activation; (c) MUC1‐C activates the BMI1 gene through MYC protein‐dependent mechanism, which affects the epigenetics of cells (David, Hamilton, & Palena, 2016; Hiraki et al., 2017); (d) MUC1‐ C could promote immune evasion and protect cells from damage through reducing the expression of PD‐L1 (Bouillez et al., 2017; Raina et al., 2015). These reports provide evidence that MUC1‐C represents a potential target for the treatment of ESCC although further mechanisms are still needed to be explored. And MUC1‐C inhibitor may become a new agent for ESCC therapy.

In conclusions based on our study, MUC1‐C affects tumor cell metabolism and alters cell apoptosis by regulating GSH levels, ROS production, and changes in mitochondrial membrane potential. All these effects are achieved through the AKT–mTORC–S6K1 signaling pathway in conjunction with TIGAR. Furthermore, targeting MUC1‐C function inhibits the cellular proliferation and survival of ESCC cells in vivo and represses the expression of TIGAR in xenograft tissues. Hence, the MUC1‐C and TIGAR may become potential targets for the treatment of ESCC cancer, and the combination therapy of the two genes may be more effective for ESCC patients.

## CONFLICTS OF INTEREST

The authors have declared that there are no conflicts of interest.

## References

[jcp27863-bib-0001] Alkhayal, K. , Abdulla, M. , Al‐obeed, O. , Kattan, W. A. , Zubaidi, A. , Vaali‐Mohammed, M. A. , … Ahmad, R. (2016). Identification of the TP53‐induced glycolysis and apoptosis regulator in various stages of colorectal cancer patients. Oncology Reports, 35(3), 1281–1286. 26675982 10.3892/or.2015.4494 PMC4750753

[jcp27863-bib-0002] Ahmad, R. , Alam, M. , Hasegawa, M. , Uchida, Y. , Alobaid, O. , Kharbanda, S. , & Kufe, D. (2017). Targeting MUC1‐C inhibits the AKT‐S6K1‐elF4A pathway regulating TIGAR translation in colorectal cancer. Molecular cancer, 16, 33. 28153010 10.1186/s12943-017-0608-9 PMC5290603

[jcp27863-bib-0003] Ahmad, R. , Raina, D. , Joshi, M. D. , Kawano, T. , Ren, J. , Kharbanda, S. , & Kufe, D. (2009). MUC1‐C oncoprotein functions as a direct activator of the NF‐κB p65 transcription factor. Cancer Research, 69(17), 7013–7021. 19706766 10.1158/0008-5472.CAN-09-0523 PMC2760979

[jcp27863-bib-0004] Ahmad, R. , Raina, D. , Trivedi, V. , Ren, J. , Rajabi, H. , Kharbanda, S. , & Kufe, D. (2007). MUC1 oncoprotein activates the IκB kinase β complex and constitutive NF‐κB signalling. Nature Cell Biology, 9(12), 1419–1427. 18037881 10.1038/ncb1661 PMC4209910

[jcp27863-bib-0005] Bouillez, A. , Rajabi, H. , Jin, C. , Samur, M. , Tagde, A. , Alam, M. , … Kufe, D. (2017). MUC1‐C integrates PD‐L1 induction with repression of immune effectors in non‐small‐cell lung cancer. Oncogene, 36(28), 4037–4046. 28288138 10.1038/onc.2017.47 PMC5509481

[jcp27863-bib-0006] Bouillez, A. , Rajabi, H. , Pitroda, S. , Jin, C. , Alam, M. , Kharbanda, A. , … Kufe, D. (2016). Inhibition of MUC1‐C suppresses MYC expression and attenuates malignant growth in KRAS mutant lung adenocarcinomas. Cancer Research, 76(6), 1538–1548. 26833129 10.1158/0008-5472.CAN-15-1804 PMC4794417

[jcp27863-bib-0007] Budanov, A. V. , Sablina, A. A. , Feinstein, E. , Koonin, E. V. , & Chumakov, P. M. (2004). Regeneration of peroxiredoxins by p53‐regulated sestrins, homologs of bacterial AhpD. Science, 304(5670), 596–600. 15105503 10.1126/science.1095569

[jcp27863-bib-0008] Bensaad, K. , Tsuruta, A. , Selak, M. A. , Vidal, M. N. C. , Nakano, K. , Bartrons, R. , … Vousden, K. H. (2006). TIGAR, a p53‐inducible regulator of glycolysis and apoptosis. Cell, 126(1), 107–120. 16839880 10.1016/j.cell.2006.05.036

[jcp27863-bib-0009] Cosentino, C. , Grieco, D. , & Costanzo, V. (2014). ATM activates the pentose phosphate pathway promoting anti‐oxidant defence and DNA repair. EMBO Journal, 30(3), 546–555. 10.1038/emboj.2010.330 PMC3034007 21157431

[jcp27863-bib-0010] David, J. M. , Hamilton, D. H. , & Palena, C. (2016). MUC1 upregulation promotes immune resistance in tumor cells undergoing brachyury‐mediated epithelial‐mesenchymal transition. Oncoimmunology, 5, e1117738. 27141403 10.1080/2162402X.2015.1117738 PMC4839328

[jcp27863-bib-0011] Dorrello, N. V. , Peschiaroli, A. , Guardavaccaro, D. , Colburn, N. H. , Sherman, N. E. , & Pagano, M. (2006). S6K1‐ and ßTRCP‐mediated degradation of PDCD4 promotes protein translation and cell growth. Science, 314(5798), 467–471. 17053147 10.1126/science.1130276

[jcp27863-bib-0012] Engelman, J. A. , Luo, J. , & Cantley, L. C. (2006). The evolution of phosphatidylinositol 3‐kinases as regulators of growth and metabolism. Nature Reviews Genetics, 7(8), 606–619. 10.1038/nrg1879 16847462

[jcp27863-bib-0013] Huang, L. , Chen, D. , Liu, D. , Yin, L. , Kharbanda, S. , & Kufe, D. (2005). MUC1 oncoprotein blocks glycogen synthase kinase 3beta‐mediated phosphorylation and degradation of beta‐catenin. Cancer Research, 65(22), 10413–10422. 16288032 10.1158/0008-5472.CAN-05-2474

[jcp27863-bib-0014] Hiraki, M. , Maeda, T. , Bouillez, A. , Alam, M. , Tagde, A. , Hinohara, K. , … Kufe, D. (2017). MUC1‐C activates BMI1 in human cancer cells. Oncogene, 36(20), 2791–2801. 27893710 10.1038/onc.2016.439 PMC5436937

[jcp27863-bib-0015] Hong, M. , Xia, Y. , Zhu, Y. , Zhao, H. H. , Zhu, H. , Xie, Y. , … Li, J. Y. (2016). TP53‐induced glycolysis and apoptosis regulator protects from spontaneous apoptosis and predicts poor prognosis in chronic lymphocytic leukemia. Leukemia Research, 50, 72–77. 27693855 10.1016/j.leukres.2016.09.013

[jcp27863-bib-0016] Joshi, M. D. , Ahmad, R. , Yin, L. , Raina, D. , Rajabi, H. , Bubley, G. , … Kufe, D. (2009). MUC1 oncoprotein is a druggable target in human prostate cancer cells. Molecular Cancer Therapeutics, 8(1), 3056–3065. 19887552 10.1158/1535-7163.MCT-09-0646 PMC2783220

[jcp27863-bib-0017] Jing, L. , Xiang, S. , Zhang, Q. , Wu , J. , Tang, Q. , Zhou, J. … Hann, S.S. (2015). Combination of curcumin and bicalutamide enhanced the growth inhibition of androgen‐independent prostate cancer cells through SAPK/JNK and MEK/ERK1/2‐mediated targeting NF‐κB/p65 and MUC1‐C. Journal of Experiment & Clinical Cancer Research, 34(1), 46. 10.1186/s13046-015-0168-z PMC4446835 25971429

[jcp27863-bib-0018] Kufe, D.W. (2013). MUC1‐C oncoprotein as a target in breast cancer; activation of signaling pathways and therapeutic approaches. Oncogene, 32(9), 1073–1081. 22580612 10.1038/onc.2012.158 PMC3621754

[jcp27863-bib-0019] Kufe, D. W. (2009). Mucins in cancer: function, prognosis and therapy. Nature Reviews Cancer, 9(12), 874–885. 19935676 10.1038/nrc2761 PMC2951677

[jcp27863-bib-0020] Leng, Y. , Cao, C. , Ren, J. , Huang, L. , Chen, D. , Ito, M. , & Kufe, D. (2007). Nuclear import of the MUC1‐C oncoprotein is mediated by nucleoporin Nup62. Journal of Biological Chemistry, 282(27), 19321–19330. 17500061 10.1074/jbc.M703222200

[jcp27863-bib-0021] Li, T. , Kon, N. , Jiang, L. , Tan, M. , Ludwig, T. , Zhao, Y. , … Gu, W. (2012). Tumor suppression in the absence of p53‐mediated cell‐cycle arrest, apoptosis, and senescence. Cell, 149(6), 1269–1283. 22682249 10.1016/j.cell.2012.04.026 PMC3688046

[jcp27863-bib-0022] Lu, Z. , Lu, F. , Zheng, Y. , Zeng, Y. , Zou, C. , & Liu, X. (2015). Grape seed proanthocyanidin extract protects human umbilical vein endothelial cells from indoxyl sulfate‐induced injury via ameliorating mitochondrial dysfunction. Renal Failure, 38(1), 100–108. 26512753 10.3109/0886022X.2015.1104609

[jcp27863-bib-0023] Ma, X. M. , & Blenis, J. (2009). Molecular mechanisms of mTOR‐mediated translational control. Nature Reviews Molecular Cell Biology, 10(5), 307–318. 19339977 10.1038/nrm2672

[jcp27863-bib-0024] Martin, J. C. , Herbert, B. S. , & Hocevar, B. A. (2010). Disabled‐2 downregulation promotes epithelial‐to‐mesenchymal transition. British Journal of Cancer, 103(11), 1716–1723. 21063401 10.1038/sj.bjc.6605975 PMC2994233

[jcp27863-bib-0025] Mizoguchi, K. , Ishiguro, H. , Kimura, M. , Takahashi, H. , Sakamoto, N. , Tanaka, T. , & Takeyama, H. (2014). Induction of apoptosis by eicosapentaenoic acid in esophageal squamous cell carcinoma. Anticancer Research, 34(12), 7145–7149. 25503142

[jcp27863-bib-0026] Mariette, C. , Piessen, G. , & Triboulet, J. P. (2007). Therapeutic strategies in oesophageal carcinoma: role of surgery and other modalities. Lancet Oncology, 8(6), 545–553. 17540306 10.1016/S1470-2045(07)70172-9

[jcp27863-bib-0027] Nath, S. , & Mukherjee, P. (2014). MUC1: A multifaceted oncoprotein with a key role in cancer progression. Trends in Molecular Medicine, 20, 332–342. 24667139 10.1016/j.molmed.2014.02.007 PMC5500204

[jcp27863-bib-0028] Ren, J. , Agata, N. , Chen, D. , Li, Y. , Yu, Wh , Huang, L. , … Kufe, D. (2004). Human MUC1 carcinoma‐associated protein confers resistance to genotoxic anticancer agents. Cancer Cell, 5(2), 163–175. 14998492 10.1016/s1535-6108(04)00020-0 PMC4217165

[jcp27863-bib-0029] Raina, D. , Ahmad, R. , Joshi, M. D. , Yin, L. , Wu, Z. , Kawano, T. , … Kufe, D. (2009). Direct targeting of the MUC1 oncoprotein blocks survival and tumorigenicity of human breast carcinoma cells. Cancer Research, 69(12), 5133–5141. 19491255 10.1158/0008-5472.CAN-09-0854 PMC2721222

[jcp27863-bib-0030] Raina, D. , Agarwal, P. , Lee, J. , Bharti, A. , McKnight, C. J. , Sharma, P. , … Kufe, D. (2015). Characterization of the MUC1‐C cytoplasmic domain as a cancer target. PLOS One, 10(8), e0135156. 26267657 10.1371/journal.pone.0135156 PMC4534190

[jcp27863-bib-0031] Raina, D. , Ahmad, R. , Rajabi, H. , Panchamoorthy, G. , Kharbanda, S. , & Kufe, D. (2012). Targeting cysteine‐mediated dimerization of the MUC1‐C oncoprotein in human cancer cells. International Journal of Oncology, 40(5), 1643–1649. 22200620 10.3892/ijo.2011.1308 PMC3326351

[jcp27863-bib-0032] Ren, J. , Bharti, A. , Raina, D. , Chen, W. , Ahmad, R. , & Kufe, D. (2006). MUC1 oncoprotein is targeted to mitochondria by heregulin‐induced activation of c‐Src and the molecular chaperone HSP90. Oncogene, 25(1), 20–31. 16158055 10.1038/sj.onc.1209012

[jcp27863-bib-0033] Raina, D. , Kosugi, M. , Ahmad, R. , Panchamoorthy, G. , Rajabi, H. , Alam, M. , … Kufe, D. (2011). Dependence on the MUC1‐C oncoprotein in non‐small cell lung cancer cells. Molecular Cancer Therapeutics, 10(5), 806–816. 21421804 10.1158/1535-7163.MCT-10-1050 PMC3092019

[jcp27863-bib-0034] R, G., Jr , Komar, A. A. , & Merrick, W. C. (2002). eIF4A: The godfather of the DEAD box helicases. Progress in Nucleic Acid Research & Molecular Biology, 72, 307. 12206455 10.1016/s0079-6603(02)72073-4

[jcp27863-bib-0035] Shi, M. , Chen, D. , Yang, D. , & Liu, X. Y. (2015). CCL21‐CCR7 promotes the lymph node metastasis of esophageal squamous cell carcinoma by up‐regulating MUC1. Journal of Experimental & Clinical Cancer Research, 34, 149. 26667143 10.1186/s13046-015-0268-9 PMC4678529

[jcp27863-bib-0036] Sonenberg, N. , & Hinnebusch, A. G. (2009). Regulation of translation initiation in eukaryotes: Mechanisms and biological targets. Cell, 136(4), 731–745. 19239892 10.1016/j.cell.2009.01.042 PMC3610329

[jcp27863-bib-0037] Shen, M. , Zhao, X. , Zhao, L. , Shi, L. , An, S. , Huang, G. , & Liu, J. (2018). Met is involved in TIGAR‐regulated metastasis of non‐small‐cell lung cancer. Molecular Cancer, 17(1), 88. 29753331 10.1186/s12943-018-0839-4 PMC5948872

[jcp27863-bib-0038] Torre, L. A. , Bray, F. , Siegel, R. L. , Ferlay, J. , Lortettieulent, J. , & Jemal, A. (2015). Global cancer statistics, 2012. CA: A Cancer Journal for Clinicians, 65(2), 87–108. 25651787 10.3322/caac.21262

[jcp27863-bib-0039] Takahashi, H. , Jin, C. , Rajabi, H. , Pitroda, S. , Alam, M. , Ahmad, R. , … Kufe, D. (2015). MUC1‐C activates the TAK1 inflammatory pathway in colon cancer. Oncogene, 34(40), 5187–5197. 25659581 10.1038/onc.2014.442 PMC4530107

[jcp27863-bib-0040] Vousden, K. H. , & Ryan, K. M. (2009). p53 and metabolism. Nature Reviews Cancer, 9(10), 691–700. 19759539 10.1038/nrc2715

[jcp27863-bib-0041] Vanhaesebroeck, B. , Stephens, L. , & Hawkins, P. (2012). PI3K signalling: The path to discovery and understanding. Nature Reviews Molecular Cell Biology, 13(3), 195–203. 22358332 10.1038/nrm3290

[jcp27863-bib-0042] Won, K. Y. , Lim, S. J. , Kim, G. Y. , Kim, Y. W. , Han, S. A. , Song, J. Y. , & Lee, D. K. (2012). Regulatory role of p53 in cancer metabolism via SCO2 and TIGAR in human breast cancer. Human Pathology, 43(2), 221–228. 21820150 10.1016/j.humpath.2011.04.021

[jcp27863-bib-0043] Wong, E. Y. L. , Wong, S. C. C. , Chan, C. M. L. , Lam, E. K. Y. , Ho, L. Y. , Lau, C. P. Y. , … Chan, A. T. C. (2015). TP53‐induced glycolysis and apoptosis regulator promotes proliferation and invasiveness of nasopharyngeal carcinoma cells. Oncology Letters, 9(2), 569–574. 25621025 10.3892/ol.2014.2797 PMC4301475

[jcp27863-bib-0044] Xin, Z. , Gongsun, X. , Shi, M. , Song, L. , Wang, Q. , Jiang, B. , & Liu, X. (2018). Inhibition of MUC1‐C entering nuclear suppresses MYC expression and attenuates malignant growth in esophageal squamous cell carcinoma. OncoTargets and Therapy, 11, 4125–4136. 30050304 10.2147/OTT.S168813 PMC6056156

[jcp27863-bib-0045] Yin, L. , Ahmad, R. , Kosugi, M. , Kawano, T. , Avigan, D. , Stone, R. , … Kufe, D. (2010). Terminal differentiation of chronic myelogenous leukemia cells is induced by targeting of the MUC1‐C oncoprotein. Cancer biology & therapy, 10(5), 483–491. 20592495 10.4161/cbt.10.5.12584 PMC3034602

[jcp27863-bib-0046] Yamamoto, M. , Bharti, A. , Li, Y. , & Kufe, D. (1997). Interaction of the DF3/MUC1 breast carcinoma‐associated antigen and beta‐catenin in cell adhesion. Journal of Biological Chemistry, 272(19), 12492–12494. 9139698 10.1074/jbc.272.19.12492

[jcp27863-bib-0047] Yin, L. , Huang, L. , & Kufe, D. (2004). MUC1 oncoprotein activates the FOXO3a transcription factor in a survival response to oxidative stress. Journal of Biological Chemistry, 279(44), 45721–45727. 15322085 10.1074/jbc.M408027200

[jcp27863-bib-0048] Yin, L. , Kufe, T. , Avigan, D. , & Kufe, D. (2014). Targeting MUC1‐C is synergistic with bortezomib in downregulating TIGAR and inducing ROS‐mediated myeloma cell death. Blood, 123(19), 2997–3006. 24632713 10.1182/blood-2013-11-539395 PMC4014842

[jcp27863-bib-0049] Yin, L. , Kharbanda, S. , & Kufe, D. (2007). Mucin 1 oncoprotein blocks hypoxia‐inducible factor 1alpha activation in a survival response to hypoxia. Journal of Biological Chemistry, 282(1), 257–266. 17102128 10.1074/jbc.M610156200

[jcp27863-bib-0050] Yin, L. , Kosugi, M. , & Kufe, D. (2012). Inhibition of the MUC1‐C oncoprotein induces multiple myeloma cell death by down‐regulating TIGAR expression and depleting NADPH. Blood, 119(3), 810–816. 22117045 10.1182/blood-2011-07-369686 PMC3265204

[jcp27863-bib-0051] Yin, L. , Li, Y. , Ren, J. , Kuwahara, H. , & Kufe, D. (2003). Human MUC1 carcinoma antigen regulates intracellular oxidant levels and the apoptotic response to oxidative stress. Journal of Biological Chemistry, 278(37), 35458–35464. 12826677 10.1074/jbc.M301987200

[jcp27863-bib-0052] Yin, L. , Tagde, A. , Gali, R. , Tai, Y. T. , Hideshima, T. , Anderson, K. , … Kufe, D. (2017). MUC1‐C is a target in lenalidomide resistant multiple myeloma. British Journal of Haematology, 178(6), 914–926. 28643330 10.1111/bjh.14801 PMC5591051

[jcp27863-bib-0053] Yin, L. , Wu, Z. , Avigan, D. , Rosenblatt, J. , Stone, R. , Kharbanda, S. , & Kufe, D. (2011). MUC1‐C oncoprotein suppresses reactive oxygen species‐induced terminal differentiation of acute myelogenous leukemia cells. Blood, 117(18), 4863–4870. 21422470 10.1182/blood-2010-10-296632 PMC3100696

[jcp27863-bib-0054] Ye, Q. , Yan, Z. , Liao, X. , Li, Y. , Yang, J. , Sun, J. , … Huang, L. (2011). MUC1 induces metastasis in esophageal squamous cell carcinoma by upregulating matrix metalloproteinase 13. Laboratory Investigation, 91(5), 778–787. 21339746 10.1038/labinvest.2011.12

[jcp27863-bib-0055] Zhao, M. , Fan, J. , Liu, Y. , Yu, Y. , Xu, J. , Wen, Q. , … Yang, L. (2016). Oncogenic role of the TP53‐induced glycolysis and apoptosis regulator in nasopharyngeal carcinoma through NF‐κB pathway modulation. International Journal of Oncology, 48(2), 756–764. 26691054 10.3892/ijo.2015.3297

